# Severe Headache and Hypertension in a Pregnant Patient

**DOI:** 10.34067/KID.0000000948

**Published:** 2026-01-29

**Authors:** Varsha Venkatakrishna, Andrew Moses

**Affiliations:** 1 Department of Internal Medicine, Lenox Hill Hospital, New York, New York; 2 Department of Nephrology, Lenox Hill Hospital, New York, New York

**Keywords:** hypertension, quality of life, imaging

## Abstract

**Figure:**
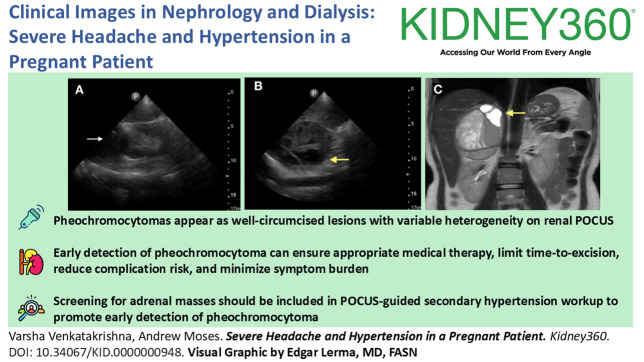

A 34-year-old female patient presented 2 months postpartum for evaluation of preeclampsia. She reported severe intermittent headache and new, labile home BP despite treatment with labetalol. BP on presentation was 131/86 mm Hg. Point-of-care ultrasound (POCUS) was performed as part of routine secondary hypertension workup and revealed a large heterogeneous mass measuring 8.86×6.88 cm bordering the right kidney with cystic loculations containing scant debris (Figure 1, A and B). Abdominal magnetic resonance imaging redemonstrated a hemorrhagic adrenal mass containing loculations with blood-fluid levels (Figure 1C). Elevated serum normetanephrine levels confirmed the diagnosis of pheochromocytoma. The patient was switched to an alpha blockade until prompt right adrenalectomy. Histopathology of the excised hemorrhagic tissue returned positive for pheochromocytoma. Symptoms and hypertension resolved postoperatively.

**Figure 1:**
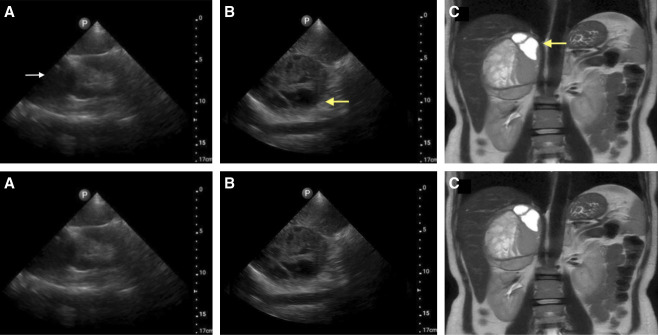
**Imaging of adrenal mass.** (A) Renal POCUS short axis view reveals a large hypoechoic mass abutting the right kidney (white arrow). (B) Renal POCUS long access view reveals the mass is well circumscribed, heterogeneous, and loculated (yellow arrow). (C) MRI redemonstrates the hemorrhagic right adrenal mass with loculated cysts containing blood-fluid levels (yellow arrow). MRI, magnetic resonance imaging.

Surveillance POCUS showed a small hypoechoic pocket of blood near the right kidney without an active bleed on Doppler.

Sonographically, pheochromocytomas appear as well-circumscribed lesions with variable heterogeneity depending on degree of internal hemorrhage or necrosis. Most tumors are solid, although hemorrhagic lesions can form complex lesions with intraluminal cysts filled with blood or necrotic debris. Pheochromocytomas >1.5 cm in size or with hemorrhage or necrosis are easiest to differentiate from adrenal parenchyma on sonography. On average, these tumors measure 5.70 cm in size and can range from 1.5 to 20.0 cm.

In a multicenter retrospective study, untreated peripartum pheochromocytoma was associated with a 27 times higher risk of severe maternal complications and fetal death. POCUS-guided screening for adrenal masses should be included in routine secondary hypertension workup to potentially reduce time to treatment, minimize complications risks, and alleviate symptom burden of pheochromocytoma.

## Teaching Points

- Pheochromocytomas appear as well-circumcised lesions with variable heterogeneity on renal POCUS.
- Early detection of pheochromocytoma can ensure appropriate medical therapy, limit time to excision, reduce complication risk, and minimize symptom burden.
- Screening for adrenal masses should be included in POCUS-guided secondary hypertension workup to promote early detection of pheochromocytoma.

